# Carbon-Supported Raney Nickel Catalyst for Acetone Hydrogenation with High Selectivity

**DOI:** 10.3390/molecules25040803

**Published:** 2020-02-13

**Authors:** Shuliang Lu, Jiajia Wu, Hui Peng, Yong Chen

**Affiliations:** SINOPEC Beijing Research Institute of Chemical Industry, Beijing 100013, China; Lusl.bjhy@sinopec.com (S.L.); wujj.bjhy@sinopec.com (J.W.); pengh.bjhy@sinopec.com (H.P.)

**Keywords:** carbon-support, Raney nickel, acetone hydrogenation, acidity

## Abstract

Catalysts with high selectivity play key roles in green chemistry. In this work, a granular Raney Ni catalyst using carbon as support (Raney Ni/C) was developed by mixing phenolic resin with Ni-Al alloy, conducting carbonization at high temperature, and leaching with alkaline liquor. The as-prepared Raney Ni/C catalyst is suitable for use in fix-bed reactors. Moreover, it shows high activity and selectivity for catalytic acetone hydrogenation. For instance, at the reaction temperature of 120 °C, the conversion of acetone can reach up to 99.9% and the main byproduct methyl isobutylcarbinol (MIBC) content can be diminished to 0.02 wt%. The Raney Ni/C may represent a new type of shaped Raney metal catalysts, which are important fix-bed catalysts in chemical industry.

## 1. Introduction

Catalysts play key roles in the chemical industry, and more than 90% of today’s chemical processes use catalysts [1,2]. Industrial catalysts have some well-defined features, e.g., activity, selectivity, stability, strength, recoverability, and poison resistance, related to the basic demands of productive process to achieve green, environmental, predictable, and economic operation [3]. Nowadays, it has been realized that the supports, used to disperse and anchor the catalytic active component, play a role in determining the catalytic properties as a result of their physical-chemical properties.

Isopropanol is a commonly used bulk chemical widely used in organic synthesis, industrial solvent, and fuel cells [4,5,6,7]. Catalytic acetone hydrogenation is a cheap, safe, and mature alternative method for isopropanol production due to its easy catalytic reduction of C=O into C-OH [8]. Currently, Raney nickel is adopted as a catalyst for the basic acetone hydrogenation process [5,8]. However, powdered Raney nickel cannot be applied in a fix-bed reactor, and is mainly employed in slurry phase reactors for batch productions. Besides Raney nickel, traditional metal/oxide catalysts are also used in acetone hydrogenation, including Ni/Al_2_O_3_ [9], Ni/CeO_2_ [10], Ni/SiO_2_ [11], and Pt/Fe_3_O_4_ [12]. Nevertheless, these tradition metal/oxide catalysts always exhibit acidity due to the intrinsic characteristics of supports. Consequently, acidity always leads to byproducts such as methyl isobutyl ketone (MIBK), methyl isobutylcarbinol (MIBC), isopropyl ether (IPE), and others [13,14,15,16,17,18,19], which require a further removal process. Additionally, the reclamation of traditional metal/oxide catalysts requires high-temperature calcination or strong acid/base dissolution, which can potentially cause environmental contamination.

Carbon materials have a variety of advantages (such as stable chemical-mechanical properties, ease with regards to reclamation [20,21,22,23,24]), and have wide applications in the metal catalysts as supports [25,26,27,28,29,30]. Moreover, the pore structures and surface properties of carbon materials can be adjusted by changing the carbon precursor, adjusting the carbonization temperature, and doping heteroatoms [31,32]. Therefore, a carbon material with weak acidity can be synthesized via the above-mentioned methods and used for supporting Raney nickel. In this case, challenges such as the difficulty of using Raney nickel powder in fixed-bed reactors, byproducts formation during the acetone hydrogenation catalyzed by acidity, and environmental contamination aroused from catalyst reclamation could be overcome. Here, a granular carbon-supported Raney nickel catalyst (Raney Ni/C) with weak acidity was prepared via mixing phenolic resin with Ni-Al alloy, conducting carbonization at high temperatures, and leaching with alkaline liquor. Then, the Raney Ni/C was characterized by Raman spectra, Scanning Electron Microscopy (SEM), Brunauer Emmett Teller (BET), and NH_3_ temperature-programmed desorption (NH_3_-TPD), respectively. Its catalytic properties in terms of acetone hydrogenation were studied at different reaction temperatures and compared with a traditional Ni/Al_2_O_3_ catalyst.

## 2. Results and Discussion

**Figure 1:**
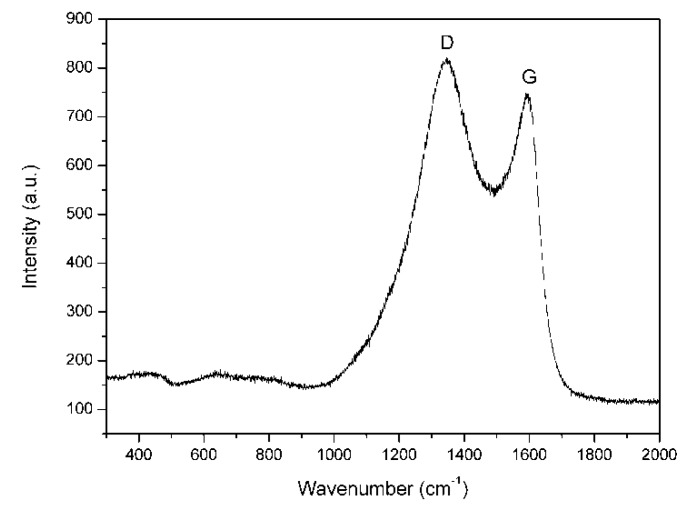
Raman spectrum of Raney Ni/C catalyst.

**Figure 2:**
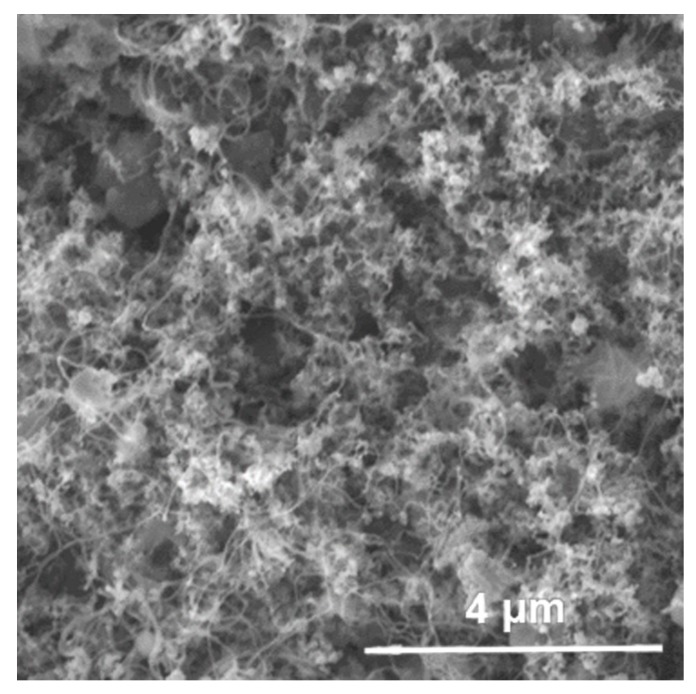
SEM image Raney Ni/C catalyst.

**Figure 3:**
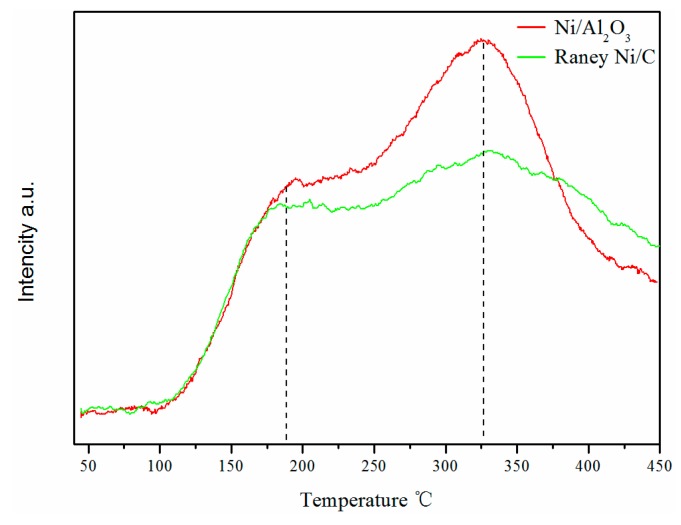
NH_3_-TPD profiles of Raney Ni/C catalyst and Ni/Al_2_O_3_ catalyst.

The microstructure of Raney Ni/C was characterized by Raman spectrum. As shown in Figure 1, there are two bands at 1340 cm^−1^ (D band) and 1590 cm^−1^ (G band), which are not separated completely. The D band corresponds to the common feature of all disordered graphitic carbon, while the G band represents the graphitic carbon phase with an sp2 electronic configuration [33]. In addition, the relative intensity of both bands reflects the degree of graphitization [34]. Here, the ID/IG of Raney Ni/C is about 3.2, which indicates a low graphitization degree and demonstrates the dominant disordered structure of the carbon support of Raney Ni/C. In the N_2_-sorption measurements, the specific area and average pore width of Raney Ni/C are determined as 111 m^2^∙g^−1^ and 3.3 nm, respectively, by virtue of BET equation; the pore volume is calculated to be 0.092 cm^3^∙g^−1^ via Barrett-Joyner-Halenda (BJH) method; and the specific area, average pore width, and pore volume of Ni/Al_2_O_3_ are 105 m^2^∙g^−1^, 10.8 nm, and 0.29 cm^3^∙g^−1^, respectively. Hence, the Raney Ni/C is mesoporous and possesses a more small-aperture pore structure than Ni/Al_2_O_3_. SEM image shown in Figure 2 illustrates the porous structure of Raney Ni/C as expected. The acidities of both Raney Ni/C and Ni/Al_2_O_3_ were measured by NH_3_-TPD and are shown in Figure 3. Both Raney Ni/C and Ni/Al_2_O_3_ show two desorption peaks. Obviously, they have similar weak acidic site desorption peaks at 180 °C; meanwhile, the Raney Ni/C shows a smaller medium-strong acidic sites desorption peak than the Ni/Al_2_O_3_ at 350 °C [15]. As proven, the acidity of Raney Ni/C is weaker than Ni/Al_2_O_3_.

The numerical results of acetone hydrogenation at 80–150 °C catalyzed by Raney Ni/C and Ni/Al_2_O_3_ are listed in Table 1. The conversion of acetone increases with increasing reaction temperature, and all reach above 99% at 120–150 °C, indicating the catalytic activity of Raney Ni/C is as high as that of commercialized Ni/Al_2_O_3_. Moreover, MIBC is the main byproduct of acetone hydrogenation. When the reaction temperature reached 150 °C, the concentration of MIBC catalyzed by Raney Ni/C was merely 500 ppm (0.05 wt%). As for Ni/Al_2_O_3_, the MIBC concentration was 56,000 ppm (5.6 wt%) at the same reaction conditions. Obviously, the Raney Ni/C catalyst can reduce the MIBC byproduct of acetone hydrogenation. This can be attributed to the different acidities of catalyst supports attached to carbon and Al_2_O_3_, respectively. Furthermore, in order to testify the effect of acidity on catalytic acetone hydrogenation, a strong acidic cation exchange resin was simply mixed with Raney Ni/C and used as an acetone hydrogenation catalyst. As shown in Table 1, the concentration of MIBC reached up to 150,000 ppm (15 wt%). The result confirms the MIBC byproduct is mainly caused by catalyst acidity and the yield of MIBC increases with the increasing catalyst acidity.

## 3. Materials and Methods

In this study, a typical Raney Ni/C was prepared as follows: 2.5 g Ni-Al alloy powder, 10 g powdery phenol formaldehyde resin, and 1.2 g hexamethylenetetramine were mixed by a high speed mixer (Zhongcheng pharmaceutical machinery, Changsha, Hunan, China). The mixture was filled into a 2 mm mold and compressed tightly at a pressure of 5 Mpa. Then, a 2 mm-thick plate was obtained. Next, the 2 mm-thick plate was dried at 150 °C for 30 min, and mechanically cut into particles with a particle size of approximately 2 mm. Finally, granular Raney Ni/C catalyst was obtained through carbonizing the 2 mm particles at the conditions of 800 °C and nitrogen atmosphere for 2 h, and aluminum of the carbonized particles was leached using alkaline liquid. The Ni-loading of granular Raney Ni/C is 20 wt%. The traditional Ni/Al_2_O_3_ catalyst was prepared according to the commercial impregnation method. To be specific, 10 g Ni(NO_3_)_2_∙6H_2_O was dissolved in 3 g deionized water, to which 10 g Al_2_O_3_ was added and impregnated for 1 h. The above mixture was dried at 120 °C for 12 h, followed with calcined at 360 °C for 4 h. Finally, Ni/Al_2_O_3_ was obtained after reduced by hydrogen at 400 °C for 8 h. The Ni-loading of Ni/Al_2_O_3_ was 20 wt%.

More detailed experimental information, including chemical materials, characterization methods, and hydrogenation experiment, is attached in the supplementary material.

## 4. Conclusions

Raney Ni is shaped as a granular catalyst by using carbon material as support. This Raney Ni/C can be used in fix-bed reactors, which overcomes the disadvantages of commonly used Raney nickel catalyst. Moreover, the Raney Ni/C can dramatically reduce the MIBC byproduct for catalytic acetone hydrogenation, as a result of the weak acidity of the carbon-support. The recycling of the Raney Ni/C would only need calcining in air to get rid of carbon-support, and the remaining metal alloys could be reused directly. We believe that the preparing method of the granular carbon-supported Raney nickel catalyst could be applied to a series of Raney metal catalysts, for instance, Raney cobalt and Raney copper, which are important fix-bed catalysts in chemical industry.

## Figures and Tables

**Table 1:** Numerical results of acetone hydrogenation catalyzed by Raney Ni/C, Ni/Al_2_O_3_, and mixed catalyst of Raney Ni/C and strong acidic cation exchange resin.

Catalyst	Temperature (°C)	Conversion of AC (%)	MIBC (wt%)
Ni/Al_2_O_3_^a^	80	94.3	0.3
	100	99.9	1.9
	120	99.9	2.6
	150	99.9	5.6
Raney Ni/C ^b^	80	76.3	0
	100	93.4	0.006
	120	99.9	0.02
	150	99.9	0.05
Resin + Raney Ni/C ^c^	100	99.9	15

Reaction conditions: ^a^ 20 g acetone, 1 g Ni/Al_2_O_3_, 3 Mpa H_2_ pressure, and magnetic stirring 10 h; ^b^ 20 g acetone, 1 g Raney Ni/C, 3 Mpa H_2_ pressure, and magnetic stirring 10 h; ^c^ 20 g acetone, 1 g strong acidic cation exchange resin, 1 g Raney Ni/C, 3 Mpa H_2_ pressure, and magnetic stirring 10 h.

## Supplementary Materials

The following are available online, chemical materials, characterization methods, and hydrogenation experiment.
