# Supplementary material for: Carbon-Supported Raney Nickel Catalyst for Acetone Hydrogenation with High Selectivity
Source: Molecules. 2020 Feb 13;25(4):803. doi: 10.3390/molecules25040803 (PMC7070612; doi:10.3390/molecules25040803)
Supplement: Supplementary file 1 [file molecules-25-00803-s001.pdf]

Supplementary material

Carbon-Supported Raney Nickel Catalyst for Acetone Hydrogenation with High Selectivity

Shuliang Lu[a], Jiajia Wu[a], Hui Peng[a], and Yong Chen*[a]
SINOPEC Beijing Research Institute of Chemical Industry, Beijing 100013, China.

1. Details of Chemical Materials Used

All chemicals were bought from different companies and used as received. Specifically, Ni-Al alloy powder (48 wt.% Ni) and Al_2O_3 were purchased from Innochem Ind. Co.; phenol formaldehyde resin (2123) was from Xinxiang Bomafengfan Company; and acetone (AR), hexamethylenetetramine (AR), and $Ni(NO_3)_2•6H_2O$ were from Tianjin Guangfu Technology Company.

2. Characterization for Raney Ni/C and Ni/Al_2O_3

The Raman spectra were recorded on a LabRam HR 800 micro Raman spectrometer (Horiba Jobin Yvon) with 532nm radiation between 400cm^{-1} and 3000cm^{-1} with a resolution of 1cm^{-1}. The surface morphologies and structures were observed on a scanning electron microscope (SEM) (S-4800, Hitachi, Japan) operated at 1kV. The Brunauer Emmett Teller (BET) specific area and pore volume were determined using Micrometeritics ASAP 2020 physical adsorption apparatus at 77K. The temperature programmed desorption of NH_3 (NH_3-TPD) studies were carried out in Micrometeritics AutoChem II 2920 with a thermal conductivity detector (TCD).

3. catalytic acetone hydrogenation experiment

20g acetone and 1g catalyst were added directly into a 50ml autoclave. Before the reaction, the autoclave was purged three times with H_2, followed by pressurizing with H_2 to 3MPa. Then, the autoclave was heated at a specific temperature and stirred magnetically at about 400rpm. Samples (0.2mL for each) were taken out after 10h by a syringe with a microfiltration head. Each sample was analyzed by gas chromatography equipped with a flame ionization detector (GC-FID, Agilent 7890, equipped with a DB-WAX capillary column 30m × 0.32mm i.d. × 0.25μm film thickness) using an external standard method.
